# Variation in the implementation of PaTz: a method to improve palliative care in general practice - a prospective observational study

**DOI:** 10.1186/s12904-020-0514-6

**Published:** 2020-01-16

**Authors:** Ian Koper, H. Roeline W. Pasman, Bart P. M. Schweitzer, Greet van der Zweep, Gon Uyttewaal, Bregje D. Onwuteaka-Philipsen

**Affiliations:** 1 Department of Public and Occupational Health, Amsterdam Public Health research institute, Amsterdam UMC, Vrije Universiteit Amsterdam, Amsterdam, The Netherlands; 2 Stichting Fibula, Utrecht, The Netherlands; 3 Academic Hospice Demeter, De Bilt, The Netherlands

**Keywords:** Multidisciplinary, Palliative care, Primary care

## Abstract

**Background:**

PaTz (palliative care at home) is a method to improve palliative care in the primary care setting in the Netherlands. PaTz has three basic principles: (1) local GPs and DNs meet at least six times per year to identify and discuss their patients with a life-threatening illness; (2) these meetings are supervised by a specialist palliative care professional; (3) groups use a palliative care register on which all identified patients are listed. Since the start in 2010, the number of PaTz-groups in the Netherlands has been growing consistently. Although the theory of all PaTz-groups is the same, the practical functioning of PaTz-groups may vary substantially, which may complicate further implementation of PaTz as well as interpretation of effect studies. This study aims to describe the variation in practice of PaTz-groups in the Netherlands.

**Method:**

In this prospective observational study, ten PaTz-groups logged and described the activities in their meetings as well as the registered and discussed patients and topics of discussions in registration forms for a 1 year follow-up period. In addition, non-participatory observations were performed in all participating groups. Meeting and patient characteristics were analysed using descriptive statistics. Conventional content analysis was performed in the analysis of topic discussions.

**Results:**

While the basic principles of PaTz are found in almost every PaTz-group, there is considerable variation in the practice and content of the meetings of different PaTz-groups. Most groups spend little time on other topics than their patients, although the number of patients discussed in a single meeting varies considerably, as well as the time spent on an individual patient. Most registered patients were diagnosed with cancer and patient discussions mainly concerned current affairs and rarely concerned future issues.

**Conclusion:**

The basic principles are the cornerstone of any PaTz-group. At the same time, the observed variation between PaTz-groups indicates that tailoring a PaTz-group to the needs of its participants is important and may enhance its sustainability. The flexibility of PaTz-groups may also provide opportunity to modify the content and tools used, and improve identification of palliative patients and advance care planning.

## Background

Palliative care is challenging care, primarily focused on multidimensional symptom relief and quality of life rather than on curation and life prolongation [1]. In the Netherlands, palliative care is provided according to a coordinated palliative care model [2], and in the primary care setting, general practitioners (GPs) and district nurses (DNs) are the designated palliative care providers [3, 4]. The provision of good palliative care requires proper communication, coordination and collaboration between healthcare providers and with patients [5–8]. Already facing a high work load [9, 10], the combination of an aging population and the Dutch policy to provide palliative care at home where possible [11], is likely to put a strain on GPs [12] and DNs [13] alike. At the same time, market mechanisms in the Dutch health care system have led to scattering of home care organisations, impeding communication and collaboration between GPs and DNs [14]. Evidently, the provision of good palliative care in the primary care setting is under threat.

In 2010, an initiative to reinforce communication and collaboration between GPs and DNs, called PaTz (acronym for ‘Pallatieve Thuiszorg’; palliative care at home) was introduced in the Netherlands [15]. Derived from the British Gold Standards Framework (GSF) [16], PaTz aims to improve palliative care in the primary care setting through timely identification of patients eligible for palliative care, improving expertise and reinforcing the collaboration and communication between key healthcare providers in the primary care setting. The basic principles of PaTz are summarized in Table 1. Recurrent interprofessional meetings between local GPs and DNs, supported by a specialist palliative care professional (physicians and nurses with formal palliative care training) are the foundation of each PaTz-group. Participants identify patients with palliative care needs using the Surprise Question [17] (SQ: would I be surprised if this person died in the coming year?), put them on a register and code the patients with a colour indicating the urgency, intensity and/or the complexity of the care needs of that patient and his or her relatives. As such, the register provides an overview of all identified patients in the PaTz-group and serves as the backbone for the meetings. Currently, two versions of the PaTz-register are in use. The first version is the original version, a simple Excel-file in which basic information regarding all patients, their diagnosis and their stability is registered in a single Excel-sheet. The second version is an extended, web-based register called the PaTz-portal in which, apart from the basic information and the colour code, members of the PaTz-group can click on a patient to open that patient’s page, where they are prompted to provide additional information regarding the patient. This includes a description of the patient’s current problems in four dimensions as well as future problems and care needs. In the PaTz-portal, other tools and interventions that may be helpful in the care for the patient are suggested, like a joint home visit of GP and DN.

**Table 1:** The three basic principles of PaTz

(1) In a PaTz-group, local GPs and DNs meet at least six times per year to identify and discuss their patients with a life-threatening illness;	
(2) PaTz-meetings are supervised by a specialist palliative care professional;	
(3) PaTz-groups use a palliative care register on which all patients with a limited life expectancy are listed.	

While clear benefits in terms of patient outcomes have yet to be determined, evaluation studies of PaTz have shown positive results. PaTz-participants feel that PaTz improves collaboration, while strengthening participants’ expertise and providing emotional support [15], and PaTz is associated with improved communication, both between healthcare providers and with patients [18]. The PaTz-register seems a crucial element in PaTz-groups, as compared to patients who are not on the register, the preferred place of death is more often known for patients who are on the register, who also are less often admitted to the hospital in the final month [19]. In addition, their death is anticipated earlier by their GP, treatment is aimed at palliation earlier and they more often have conversations on end of life topics, like life expectancy and palliative care treatment options [19].

Since the first PaTz-groups in 2010, over 180 PaTz-groups have been established throughout the Netherlands [20]. Before the start of a new PaTz-group, the PaTz-foundation provides training for the chair and, if needed, the Comprehensive Cancer Centre Netherlands (IKNL) provides a specialist palliative care professional [21]. But from that moment on the group is left without regulation from outside, and although the three principles are the basis for any PaTz-group, there are some practical examples of variation between PaTz-groups in composition and use of additional elements. For example, there are groups in which a coordinator of volunteers in palliative care joins the meetings, while in other groups a spiritual caregiver is present. Also, some groups use the original PaTz-register, while others have switched to the web-based version, or have been using this version from the start. Thus, while the theory of PaTz is known, the functioning of PaTz-groups in practice remains unclear. A clear perspective on the extent of this variation is primarily important for further implementation and development strategies. Secondly, uncertainty regarding the variation in practice of PaTz-groups complicates interpretation of studies on the effect of PaTz-groups. Therefore, this study aims to describe the practice of PaTz-groups by investigating how the basic principles of PaTz are applied in practice, and what the content of PaTz-meetings is.

## Methods

### Design

To investigate the practice of running PaTz-groups in the Netherlands we used a prospective observational design. For a follow-up period of 1 year, chairs of participating PaTz-groups were asked to log and describe the activities in their meetings. In addition, non-participatory observations were performed in all participating groups. A mortality follow-back design was used to register the date of death of patients in included PaTz-groups.

### Recruitment of participants

**Fig. 1:**
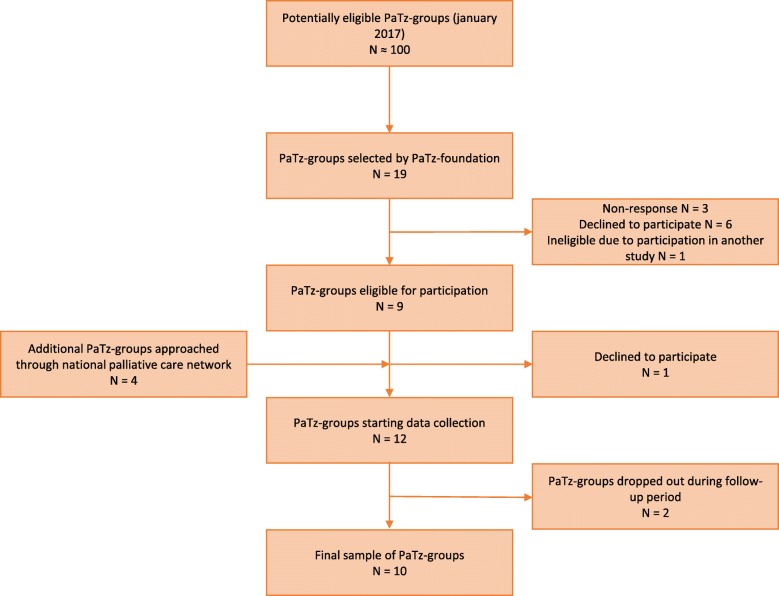
Flowchart of inclusion of PaTz-groups

Recruitment of PaTz-groups took place between January 2017 and September 2017. PaTz-groups were eligible for inclusion if they had been running for a year or longer, and were not participating in another study, influencing their performance. At that time, approximately 100 PaTz-groups were eligible for inclusion. At first, PaTz-groups were recruited through contacts at the PaTz-foundation, who provided a list of 19 PaTz-groups who might be interested in participation. The chairs of these 19 groups were sent an information letter regarding the content of study and asked whether the group was interested in participating. Non-responding groups were sent a reminder once. Three groups responded to neither the initial invitation nor the reminder, six groups refused to participate and one group was already participating in a conflicting study. The nine remaining groups agreed to participate. In an attempt to add more groups to the sample, we issued a call to participate through the umbrella organisation of palliative care networks in the Netherlands (Fibula), which provided a list of four other groups that were interested in participation. A member of the research team visited these groups to explain the study and three groups agreed to participate, adding up to a total sample of 12 PaTz-groups. Although the observations were performed in all 12 groups, one group stopped participating after the observations, and one group did not start registration at all. In the end, 10 PaTz-groups completed registration in the follow-up period and were reimbursed for their efforts. Fig. 1 summarizes the inclusion in a flowchart.

### Data collection

Data collection took place between January 2017 and November 2018. Chairs were asked to register who were present at the meetings, which patients were put on the PaTz-register, and which patients were discussed. They were also asked to describe what topics were covered in the discussions, both regarding the patients as well as general topics and how much time was spent on each subject. A form was created to guide the chairs through the registration of activities (see Additional file 1) and reminders were sent to chairs who did not return the forms. The first two meetings, a researcher performed non-participatory observations using a topic list (see Additional file 2), paying extra attention to the basic principles of PaTz in each PaTz-group and the interaction between members of the group. After the follow-up period, a researcher (IK) visited the GPs of the participating PaTz-groups to assess which patients on the PaTz-register had died in that period, and registered their date of death.

### Data analysis

Analysis of meeting characteristics derived from the non-participatory observations and registration forms and patient characteristics derived from the registration forms were performed using descriptive statistics. Selection of specific groups of patients was performed where appropriate. The descriptions of topics in patient discussions were analysed by IK and discussed with RP and BOP using conventional content analysis, in which codes and categories are derived from data during the analysis rather than established beforehand [22]. Two thirds (248/384) of the topic descriptions contained information eligible for analysis. During the first analysis, the codes and categories that were generated from the data underwent content and definition changes, resulting in a final coding tree consisting of three categories: current or future situation, the content of the discussion and the domain of discussed situation. This coding tree was discussed with RP and BOP before using in a second analysis of the data. In the second analysis new codes were added for data that did not fit into an existing code.

Meeting and patient characteristics were compared between the groups to establish practice variation, while the topics of patient discussion were analysed collectively to describe their general content.

## Results

Of the 10 PaTz-groups that completed follow-up and mortality follow-back, four groups were situated in a major city, five in a town or suburb and one group was situated in a rural area. At the time of inclusion, seven groups had been active for more than 5 years, two for more than 3 years, and one group had been active for just over a year.

### Completeness of data

In all 10 groups, observations of two meetings were performed. Regarding the registration data, there was one PaTz-group that did not return a registration form for all meetings, due to absence of the chair on one occasion. In total, 78 out of a possible 79 registration forms were included in the analysis. Unfortunately, data on the (changes in) colour coding of registered patients was insufficiently reported and could not be analysed.

### Application of basic principles of PaTz

From the observations and registration we found that in all groups but one, the basic principles of PaTz were met. The core of all but one groups consisted of GPs, DNs and a specialist palliative care professional, all groups used a PaTz-register, whether it be the original Excel-sheet or the more advanced PaTz-portal, and all groups met at least six times per year.

One particular group stood out as GPs were not the driving force of this group. The backbone of this unique group consisted of palliative care specialists from an academic hospice and local DNs, while GPs only joined the meeting when deemed necessary, by the chair’s invitation. This hospice-centred group also had a different meeting frequency, as this group met once every 2 weeks, while we found that the other groups met once every 6–8 weeks. The specialist palliative care professionals in the different groups fulfilled their role diversely. Some experts kept a low profile, only giving advice or information when asked directly. Others were more involved, and one specialist palliative care professional, albeit unofficially, even took over chairmanship of a meeting, deciding which patients to discuss and elaborating on palliative care subjects without being asked. Some groups planned their meetings after working hours, other groups met during lunch time, whichever was preferred by the group members. In most groups, healthcare providers from other disciplines, like spiritual caregivers, a coordinator of palliative care volunteers and nurse specialists, joined the meetings, and observations showed that they incidentally contributed to patient discussions by asking questions, expressing their view on a subject or proposing involvement in a patient.

We found that in all groups a palliative care register was used to list patients with a limited life expectancy. In the groups using the PaTz-portal, we observed that the PaTz-portal provided guidance and structure when discussing a patient. The hospice-centred group used a custom online register, and for each patient a separate online care plan, featuring a four-dimensional description of the patient, his or her problems and wishes and care, was filled out. An overview is presented in Table 2.

**Table 2:** Application of the basic principles of PaTz in 10 PaTz-groups during a one-year follow-up period

PaTz-group	1. Group composition			2. Use of PaTz-register	3. Meeting frequency	Basic principles met?
	Specialist palliative care professional	GP	DN			
1	Yes, a GP	Yes	Yes	Excel-sheet	Once per 2 months	Yes
2	Yes, a GP	Yes	Yes	Excel/PaTz-Portal	Once per 2 months	Yes
3	Yes, a GP and a nurse specialist	Yes	Yes	Excel/PaTz-Portal	Once per 2 months	Yes
4	Yes, a hospital nurse	Yes	Yes	Excel-sheet	Once per 2 months	Yes
5	Yes, a GP and a nurse specialist	Only on request	Yes	Custom version of register	Twice per month	No
6	Yes, a GP and a nurse specialist	Yes	Yes	PaTz-Portal	Once per 2 months	Yes
7	Yes, an elderly care specialist and a nurse specialist	Yes	Yes	PaTz-Portal	Once per 2 months	Yes
8	Yes, an elderly care specialist and a nurse specialist	Yes	Yes	PaTz-Portal	Once per 2 months	Yes
9	Yes, a GP	Yes	Yes	Excel/PaTz-Portal^a^	Once per 2 months	Yes
10	Yes, a GP	Yes	Yes	Excel-sheet	Once per 2 months	Yes

^a^During the follow-up period this group switched to the PaTz-portal

### Content of PaTz-meetings

#### Meeting characteristics

An overview of the characteristics of the meetings of the participating PaTz-groups during the follow-up period can be found in Table 3. It shows that all groups but the hospice-centred group met 5–7 times in the follow-up period. Generally, the groups were made up of 4–6 GPs, 3 or 4 DNs, 1 consultant in palliative care and 1 additional discipline, varying from a coordinator of volunteers in palliative care to a spiritual caregiver or a nurse elderly care specialist. From the observations we found that while all PaTz-groups were on a first-name basis, the interaction between participants varied across groups, likely depending on their personalities and familiarity with each other. Consistent throughout all groups, however, was the seeming reluctance of DNs to introduce a patient for discussion or engage in patient discussions started by others.

**Table 3:** Characteristics of meetings of 10 PaTz-groups in a one-year follow-up period, derived from registration data

	Total	Group 1	Group 2	Group 3	Group 4	Group 5	Group 6	Group 7	Group 8	Group 9	Group 10
Group composition (number present on average)											
GPs	5	6	5	6	6	0^a^	3	6	4	6	5
DNs	3	3	3	3	2	4	2	4	4	3	4
Consultant in palliative care	1	1	1	1	1	2	2	2	2	1	1
Other disciplines	1	1	0^a^	1	0^a^	2	2	0^a^	1	1	1
Number of meetings	79	6	5	6	6	25	7	6	6	7	5
Number of patients on the PaTz-register	583	75	107	122	48	40	45	29	39	36	42
Patient discussions											
Individual patients discussed	243 (42%)	39 (52%)	13 (12%)	21 (17%)	23 (48%)	39 (98%)	22 (49%)	21 (72%)	27 (69%)	26 (72%)	12 (29%)
Number of times individual patients were discussed											
Once	163/243 (67%)	33 (85%)	10 (77%)	19 (91%)	16 (70%)	10 (26%)	12 (55%)	18 (86%)	18 (67%)	20 (77%)	7 (58%)
Twice	54/243 (22%)	5 (13%)	3 (23%)	2 (9%)	7 (30%)	10 (26%)	8 (36%)	2 (10%)	6 (22%)	6 (23%)	5 (42%)
Three or more times	26/243 (11%)	1 (3%)	0	0	0	19 (49%)	2 (9%)	1 (5%)	3 (11%)	0	0
Discussed only after death	58/243 (24%)	4 (10%)	6 (46%)	12 (57%)	3 (13%)	1 (3%)	11 (50%)	8 (38%)	12 (44%)	0	1 (8%)
Number of patient discussions per meeting (mean, range)	4.8 (0–20)	7.7 (0–20)	3.2 (1–5)	3.8 (2–7)	5.0 (3–7)	4.8 (0–10)	5.0 (1–11)	4.2 (1–6)	6.7 (4–14)	4.6 (3–6)	3.4 (1–6)
Duration per patient											
Mean (range), minutes	8.8 (1–45)	3.5 (1–8)	13 (3–40)	15 (3–45)	11 (5–20)	11 (1–20)	9 (3–20)	8 (1–20)	5 (3–16)	12 (1–20)	8 (5–15)
25–75%, minutes	5–10	3–4	3–18	5–23	5–13	8–12	5–12	3–10	5–5	9–15	5–10
Discussion of other topics											
Number of other topics discussed	94	4	13	3	17	5	24	6	12	6	4
Minutes spent discussing other topics per meeting (mean, range)	5.7 (0–90)	2.5 (0–5)	18.1 (0–90)	3.3 (0–10)	3.6 (5–20)	3.4 (0–10)	2.5 (5–15)	5.8 (0–15)	3.6 (1–20)	7.5 (0–10)	3.8 (0–10)

^a^less than 0,5 present on average

Table 3 shows that the mean number of individual patients that was discussed per group during the follow-up period was 24 (42% of all patients on the register), ranging from 13 (or 12%) to 39 (or 98%). The majority of discussed patients were discussed once, and one quarter was only discussed after death. Regarding group composition, the proportion of patients discussed and frequency they were discussed, the hospice-centred group is a clear outlier here.

The number of patients discussed and associated amount of time spent per patient varies both between and within all groups. Some groups averaged less than 4 patients per meeting, while others averaged more than 6, and while some groups never spent more than 15 min discussing an individual patient, other groups spent 40 min or more. The number of patients discussed and time spent on individual patients also varied per meeting within the same group, as can be seen by the ranges displayed in Table 3. Interestingly, two groups had one meeting were no patients were discussed: group 1 and group 5. Further enquiry revealed that group 1 had dedicated that meeting to cooperation with local pharmacists and had spent the entire meeting on this topic, while group 5 simply had no patients to discuss at that time.

Finally, Table 3 shows that the time spent on the discussion of other topics differed greatly between groups, and between meetings. While some groups spent 2.5 min on average on other topics beside patients, there is one group that spent 18.1 min on other topics on average. At the same time, in this group, the time spent on other topics ranges from 0 to 90 min.

#### Patient characteristics

In Table 4 the characteristics of patients that were registered and patients that were also discussed in the follow-up period are displayed. On average, patients on the register were 74 years old, ranging from 34 to 101 years and 50% was male. The majority was diagnosed with cancer, and a minority was diagnosed with either organ failure or frailty/dementia. Ten percent had a different diagnosis. From the observations, it was not always clear how individual patients had been identified, but in general all PaTz-groups seemed to use the Surprise Question to identify patients with palliative care needs. Communication from the treating clinical specialist that curative treatment options had been exhausted also seemed an important identifying trigger.

**Table 4:** Characteristics of patients who were registered on the PaTz-register and who were also discussed in 10 PaTz-groups in a one-year follow-up period

Patients who were registered											
	Total N = 583	1 N = 75	2 N = 107	3 N = 122	4 N = 48	5 N = 40	6 N = 45	7 N = 29	8 N = 39	9 N = 36	10 N = 42
Age, years (mean, range)	74 (34–101)	77 (39–98)	78 (47–99)	78 (34–101)	66 (36–89)	74 (55–97)	71 (41–97)	70 (43–88)	71 (37–95)	72 (58–90)	67 (40–95)
Sex (% male)	50	49	50	49	55	43	51	66	40	59	51
Diagnosis											
Cancer %	65	36	63	57	81	78	89	100	72	58	71
Organ failure %	17	19	19	30	13	23	7	0	10	6	12
Frailty / dementia %	10	28	13	12	2	0	4	0	3	0	5
Other %	10	28	7	8	0	3	4	0	5	28	7
Patients who were also discussed											
	Total N = 243	1 N = 39	2 N = 13	3 N = 21	4 N = 23	5 N = 39	6 N = 22	7 N = 21	8 N = 27	9 N = 26	10 N = 12
Age, years (mean, range)	73 (37–98)	78 (39–98)	79 (63–94)	74 (37–95)	67 (46–81)	75 (55–97)	71 (41–92)	73 (58–88)	70 (37–90)	75 (58–90)	62 (49–92)
Sex (% male)	54	49	54	62	73	41	50	71	54	52	55
Diagnosis											
Cancer %	70	39	69	76	87	77	86	100	59	58	83
Organ failure %	13	18	23	14	13	21	5	0	15	8	0
Frailty / dementia %	6	26	8	5	0	0	0	0	4	0	8
Other %	12	28	15	5	0	3	9	0	7	35	0

A few striking differences can be seen between PaTz-groups. The mean age of registered patients ranged from 66 years to 78 years, and while most groups also registered patients with organ failure or frailty/dementia as primary diagnosis, one group only registered patients with cancer. Further, the proportion of male patients on the register also varied per group, ranging from 40 to 66%. For the patients that were also discussed (243/583), we found similar figures and variation: their mean age was 73 years, 70% was diagnosed with cancer, varying from 39 to 100% between groups. Interestingly, most groups discussed more men than women during the follow-up period (49–73%).

### Topics of patient discussions

The analysis of the topics of the patients discussions was based on 248 descriptions. An overview can be found in Table 5. More than half (139/248) of the discussions of patients occurred less than 3 months before death, while one fifth (53/248) of the discussions occurred more than 3 months before death, and another fifth (56/248) occurred after death. While we found no major differences in the topics of discussion pre or post 3 months before death, the topics of discussion after death logically mainly concerned evaluation of care. We found that the majority of discussions pre-mortem concerned current problems, treatment or wishes, mainly in the physical domain although none of the other domains are completely ignored. A relatively small proportion of the discussions concerned future situations. An overview can be found in Table 5 and some exemplary descriptions of patient discussions with their assigned codes is provided in Additional file 3.

**Table 5:** Content of patient discussions derived from informative descriptions

	Total *N* = 248	More than three months before death or end of follow-up *N* = 53	Less than three months before death or end of follow-up *N* = 139	After death *N* = 56
Discussion concerned:				
Past situation	51 (21%)	–	–	51 (91%)
Current situation	181 (73%)	48 (91%)	127 (91%)	6 (11%)
Future situation	28 (11%)	8 (15%)	18 (13%)	2 (4%)
Content of discussed situation:				
Problems	134 (54%)	37 (70%)	91 (66%)	6 (11%)
Treatment (options)	70 (28%)	19 (36%)	39 (28%)	12 (21%)
Wishes of patient/family	61 (25%)	16 (30%)	35 (25%)	10 (18%)
Evaluation of care	51 (21%)	–	–	51 (91%)
Domain of discussed situation:				
Physical	103 (42%)	29 (55%)	62 (45%)	12 (21%)
Psychological	45 (18%)	10 (19%)	30 (22%)	5 (9%)
Social	59 (24%)	12 (23%)	34 (25%)	13 (23%)
Existential	31 (13%)	7 (13%)	21 (15%)	3 (5%)
Practical	44 (18%)	14 (26%)	21 (15%)	9 (16%)
Healthcare provider – patient relationship	10 (4%)	3 (6%)	7 (5%)	–

### Other topics

Table 3 showed that, although the number of other topics discussed differed greatly per group and per meeting, all groups spent time on other topics, beside discussing patients. From the descriptions of the discussion of these topics we derived a number of categories of topics that were addressed. A large part of the discussions concerned [1] collaboration with other healthcare providers, like pharmacists and spiritual caregivers, or healthcare institutions, like local hospitals or hospices [2]; specific illnesses, treatment or medication, following from but unrelated to a specific patient, like the suitability of certain medication in palliative sedation. Other discussions concerned [3] the functioning of the PaTz-group [4]; difficult (situations regarding) patients, such as patients or families with demanding attitudes [5]; options in palliative care, such as the option to involve volunteers in palliative care; and [6] tools and knowledge centres that can be of help in palliative care.

## Discussion

### Summary of the results

While the basic principles are found in every PaTz-group but the hospice-centred group, there is considerable variation in the practice and content of the meetings of different PaTz-groups. Most groups spend little time on other topics than their patients, although the number of patients discussed in a single meeting varies considerably as does the time spent on an individual patient. Most registered patients were diagnosed with cancer and patient discussions mainly concerned current affairs and rarely concerned future issues.

### Strengths and limitations

A strength of this study is that through the combination of registration and observations, we experienced the functioning of PaTz-groups first-hand in addition to the complete picture we received from the registration. A limitation of this study lies in the fact that we included only 10 of approximately 180 PaTz-groups in the Netherlands. Considering that they were willing to participate in research, it is possible that they perform better than the average PaTz-group, regarding attendance and registration. Another weakness of the study is that one third of the topic descriptions was not informative, and the number of informative descriptions differed per group, with a large part of the informative descriptions coming from the hospice-centred group (110/248). But, as we found no major differences in topics between this group and the other groups, and the purpose of the analysis of these descriptions was to create an overview of topics in the patient discussion in general and not to compare topics between groups, we feel that the impact is limited.

### Reflections on the application of the basic principles of PaTz

All groups consist of GPs, DNs and a specialist palliative care professional, use a palliative care register and meet at least six times per year. The hospice centred-group is the exception, as GPs are not the driving force of this group and join only incidentally when invited by the hospice team, and the group meets fortnightly. This group runs by a different model, hospice care at home (HaHo), which incorporated the element of recurrent meetings from the PaTz-model as the second of four components [23]. The first component is a GP requested home visit to a patient from a hospice nurse consultant (HNC), who performs a multidimensional assessment, develops a care plan and provides specialist support to patients and relatives. The third and fourth component are telephone backup provided by the hospice and the assignment of one coordinator of care respectively [23]. As a lack of time is considered the most important barrier to participate in a regular PaTz-group [18], the higher meeting frequency may explain the practical absence of GPs in this group. The deviant group composition and the application of the HaHo-model suggest that this group cannot be seen as a regular PaTz-group. Whether it is both desirable and feasible to diffuse this model throughout the country deserves further empirical study.

How specialist palliative care professionals fulfilled their role varied across the PaTz-groups. While the added value of their knowledge and expertise is clear [15], incongruence between the specialist palliative care professional’s style and the PaTz-group’s needs and preference may cause friction and dissatisfaction. Finally, it is also worth noting that some PaTz-groups include other disciplines, like a spiritual caregiver, a volunteer in palliative care or a nurse specialist in mental health, but we could not determine whether this influenced the topics discussed.

### Reflections on the content of the meetings of the PaTz-groups

The number of patients on the register during follow-up varied greatly between PaTz-groups, ranging from 29 to 122. Although this variation may be due to differences in patient population, a more likely explanation is that different groups have different registration practice. Considering that in the three groups with highest number of patients, the patients also have the highest mean age, it could be that some groups use the SQ to include all patients who they think might die in the coming year, including all elderly patients, while other groups only register patients who are certain to die due to advanced illness in the coming year. In general, GPs find the timely identification of palliative non-cancer patients particularly challenging [24, 25], and PaTz-groups appear to be no exception. As cancer was the cause of death in 30% of all deaths in the Netherlands in 2017 and 2018 [26], the proportion of cancer patients among the registered and discussed patients is remarkably high, ranging from 36% up to a notable 100%. At the same time, it is worth noting that these patients were also more often identified closer to death. While this may explained by the typical illness trajectories [27] of these patients, late identification leaves little room for anticipatory action [28]. In addition, as these results reflect the poor performance of the SQ in the identification of non-cancer patients reported in previous literature [17], it might be worthwhile to investigate the performance of other identification tools like SPICT [29] or RADPAC [30] in this context.

Regarding the proportion of registered patients that are also discussed, we found that the hospice-centred group discussed 98% of their registered patients, which is inherent to the HaHo-method described earlier. In PaTz-groups this proportion ranges from 12 to 72%, influenced by both the groups’ registration practice, as well as their differences in discussion practice, as is shown in Table 3. While some groups seem to briefly touch upon all patients of interest and discuss on average nearly 8 patients in a single meeting, other groups seem to select a few patients per meeting to discuss more in-depth. It is also worth noting that one in four discussed patients was only discussed after death. While undoubtedly valuable and informative, this does not seem to match with one of the prime goals of PaTz, looking ahead and planning care in advance. The same applies to the topics of patient discussions which mostly concerned current affairs and rarely future situations. While the abundance of discussions of current situations probably benefits participants and possibly also future patients, it also shows that there is room for improvement regarding advance care planning.

Further, we saw that although problems and treatment options in the physical domain are the predominant subject, problems in the psychosocial and spiritual domain are also discussed. As previous research shows that patients’ and carers’ psychosocial and spiritual needs are frequently unmet in home-based palliative care [31], this is an encouraging finding. At the same time, this does not necessarily imply additional and sufficient attention for these domains in every patient that needs it. We recommend examining the added value of discussing a patient in a PaTz-group on the psychological, existential and the social domain in future studies.

### Reflections on the variation between PaTz-groups

Overall, this study shows that, even though the basics are the same, the structure and content of PaTz-groups can be adapted to the preferences of the group members. As healthcare providers in all settings, including primary care, generally have a high workload and are pressed for time, multidisciplinary meetings like PaTz need to provide value. Tailoring the structure and content of the meetings to their needs and wishes is likely to increase the perceived added value, thus increasing its sustainability [32]. In addition, the apparent flexibility of PaTz-groups introduces opportunities to improve the performance of the PaTz-groups regarding palliative patient identification and advance care planning.

As mentioned earlier, the GSF was the basis for the PaTz-method in the Netherlands. The original GSF programme required general practices to identify and register patients with a life-threatening illness and discuss these patients in quarterly team meetings [33]. Reported shortcomings of this programme included a tendency ‘to focus on mainly patients with cancer and most only in the final weeks or days of life’ [33]. Although the latter does not seem to apply to PaTz-groups, we saw that similarly, in PaTz-groups the focus lies on patients with cancer, leaving patients with other diseases overlooked. Since the start, the GSF developed ‘silver’ and ‘gold’ levels of the programme, which involve considerably more training and tools, and require more time and commitment from its participants. These ‘upgrades’ are reported to result in an increase of registered patients both with and without cancer, more patient-focused care including advance care planning and improved active support for informal caregivers [33]. It may be worthwhile to investigate whether upgrading PaTz-groups in such a fashion is both feasible and beneficial for participants and patients.

## Conclusion

Although the foundation of all PaTz-groups is the same, there is considerable variation in practical implementation of PaTz-groups, regarding organization, number and types of patients on the register and discussed during meetings between PaTz-groups. While the basic principles are essential in the functioning of PaTz-groups, the variation between PaTz-groups is also important, as tailoring a PaTz-group to the needs of its participants is likely to enhance its sustainability. The flexibility of PaTz-groups also provides ample opportunity to modify the content and tools used, and improve identification of palliative patients and advance care planning.

## Supplementary information

**Additional file 1.** Registration form part 1–4.

**Additional file 2.** Observation form – topic list.

**Additional file 3.** Exemplary descriptions of patient discussions with assigned codes.

## Data Availability

The dataset used and analysed during the current study are available from the corresponding author on reasonable request.

## Abbreviations

DN: District nurse
GP: General practitioner
GSF: Gold Standards Framework
HaHo: Hospice Assist at Home
PaTz: Palliatieve thuiszorg (palliative care at home)
