# Supplementary material for: Variation in the implementation of PaTz: a method to improve palliative care in general practice - a prospective observational study
Source: BMC Palliat Care. 2020 Jan 16;19:10. doi: 10.1186/s12904-020-0514-6 (PMC6966787; doi:10.1186/s12904-020-0514-6)
Supplement: Supplementary file 1 — Additional file 1. Registration form part 1–4. [file 12904_2020_514_MOESM1_ESM.docx]

**Registration form**

Part 1: Which patients are on the register? *(this form needs to be filled out only once!)*

| Patient (code) | Date of birth/gender | Diagnosis | Colour code/status |
| --- | --- | --- | --- |
| 01 |  |  |  |
| 02 |  |  |  |
| 03 |  |  |  |
| 04 |  |  |  |
| 05 |  |  |  |
| 06 |  |  |  |
| 07 |  |  |  |
| 08 |  |  |  |
| 09 |  |  |  |
| 10 |  |  |  |
| 11 |  |  |  |
| 12 |  |  |  |
| 13 |  |  |  |
| 14 |  |  |  |
| 15 |  |  |  |
| 16 |  |  |  |
| 17 |  |  |  |
| 18 |  |  |  |
| 19 |  |  |  |
| 20 |  |  |  |
| 21 |  |  |  |
| 22 |  |  |  |
| 23 |  |  |  |
| 24 |  |  |  |
| 25 |  |  |  |
| 26 |  |  |  |
| 27 |  |  |  |
| 28 |  |  |  |
| 29 |  |  |  |
| 30 |  |  |  |
| 31 |  |  |  |
| 32 |  |  |  |
| 33 |  |  |  |
| 34 |  |  |  |
| 35 |  |  |  |
| 36 |  |  |  |
| 37 |  |  |  |
| 38 |  |  |  |
| 39 |  |  |  |
| 40 |  |  |  |

**Registration form**

Part 2: Which patients were added/mutated in the register this meeting?

| Patient (coded) | Date of birth / gender | Diagnosis | Colour code/status | New? (Y/N) |
| --- | --- | --- | --- | --- |
|  |  |  |  |  |
|  |  |  |  |  |
|  |  |  |  |  |
|  |  |  |  |  |
|  |  |  |  |  |
|  |  |  |  |  |
|  |  |  |  |  |
|  |  |  |  |  |
|  |  |  |  |  |
|  |  |  |  |  |
|  |  |  |  |  |
|  |  |  |  |  |
|  |  |  |  |  |
|  |  |  |  |  |
|  |  |  |  |  |
|  |  |  |  |  |
|  |  |  |  |  |
|  |  |  |  |  |
|  |  |  |  |  |
|  |  |  |  |  |
|  |  |  |  |  |
|  |  |  |  |  |
|  |  |  |  |  |
|  |  |  |  |  |

Part 3: Discussed patients (topics and duration)

| Patient (coded): |  |
| --- | --- |
| Topic(s): | Time (min): |

| Patient (coded): |  |
| --- | --- |
| Topic(s): | Time (min): |

| Patient (coded): |  |
| --- | --- |
| Topic(s): | Time (min): |

| Patient (coded): |  |
| --- | --- |
| Topic(s): | Time (min): |

Part 3: Discussed patients (topics and duration)

| Patient (coded): |  |
| --- | --- |
| Topic(s): | Time (min): |

| Patient (coded): |  |
| --- | --- |
| Topic(s): | Time (min): |

| Patient (coded): |  |
| --- | --- |
| Topic(s): | Time (min): |

| Patient (coded): |  |
| --- | --- |
| Topic(s): | Time (min): |

Other topics of discussion (e.g. regarding collaboration, training, or palliative care topics in general)

| 1 |
| --- |
| 2 |
| 3 |
| 4 |
| 5 |
| 6 |
| 7 |
| 8 |
| 9 |

Registration form part 4: Presence of participants.

| Name | Discipline | Meeting 1 | 2 | 3 | 4 | 5 | 6 |
| --- | --- | --- | --- | --- | --- | --- | --- |
| 1 |  |  |  |  |  |  |  |
| 2 |  |  |  |  |  |  |  |
| 3 |  |  |  |  |  |  |  |
| 4 |  |  |  |  |  |  |  |
| 5 |  |  |  |  |  |  |  |
| 6 |  |  |  |  |  |  |  |
| 7 |  |  |  |  |  |  |  |
| 8 |  |  |  |  |  |  |  |
| 9 |  |  |  |  |  |  |  |
| 10 |  |  |  |  |  |  |  |
| 11 |  |  |  |  |  |  |  |
| 12 |  |  |  |  |  |  |  |
| 13 |  |  |  |  |  |  |  |
| 14 |  |  |  |  |  |  |  |
| 15 |  |  |  |  |  |  |  |
| 16 |  |  |  |  |  |  |  |
| 17 |  |  |  |  |  |  |  |
| 18 |  |  |  |  |  |  |  |
| 19 |  |  |  |  |  |  |  |
| 20 |  |  |  |  |  |  |  |
| 21 |  |  |  |  |  |  |  |
| 22 |  |  |  |  |  |  |  |
| 23 |  |  |  |  |  |  |  |
| 24 |  |  |  |  |  |  |  |
| 25 |  |  |  |  |  |  |  |
| 26 |  |  |  |  |  |  |  |
| 27 |  |  |  |  |  |  |  |
| 28 |  |  |  |  |  |  |  |
