# Supplementary material for: Variation in the implementation of PaTz: a method to improve palliative care in general practice - a prospective observational study
Source: BMC Palliat Care. 2020 Jan 16;19:10. doi: 10.1186/s12904-020-0514-6 (PMC6966787; doi:10.1186/s12904-020-0514-6)
Supplement: Supplementary file 2 — Additional file 2. Observation form – topic list. [file 12904_2020_514_MOESM2_ESM.docx]

**Observation form**

Main question: to what extent are the basic principles and procedures as described in theory visible in practice?

| Organisation and procedures | |
| --- | --- |
| How many participants, what distribution? |  |
| Is there a recognizable method of working? |  |
| How is the meeting built up?  Is the order of business available beforehand? |  |
| How much time is invested in the different parts? |  |
| Is a register being used? |  |
| Which other tools are used? |  |
| Who is the chair? |  |
| Who organised the meeting? |  |
| Is there training? How much time is spent on training? What subject? Is the training structural? |  |
| Points of interest regarding process or procedures. |  |
| **Time allocation and role division** | |
| How much time is spent on the different points of order? Only patients or are other topics also discussed? |  |
| How much time is spent on the different patients? |  |
| How are patients identified? Surprise Question?  Which type of patients are discussed? |  |
| What role do the participants have? Who is active, who is not? |  |
| What is the contribution of the different participants? |  |
| Points of interest regarding communication, division of roles. |  |
|  |  |
