# Supplementary material for: Variation in the implementation of PaTz: a method to improve palliative care in general practice - a prospective observational study
Source: BMC Palliat Care. 2020 Jan 16;19:10. doi: 10.1186/s12904-020-0514-6 (PMC6966787; doi:10.1186/s12904-020-0514-6)
Supplement: Supplementary file 3 — Additional file 3. Exemplary descriptions of patient discussions with assigned codes. [file 12904_2020_514_MOESM3_ESM.docx]

Additional file 3. Exemplary descriptions of patient discussions with assigned codes.

| Exemplary descriptions of patient discussions with assigned codes | | |
| --- | --- | --- |
| **Group number and patient characteristics** | **Description of discussion** | **Coded as discussion of:** |
| Group 2 discussing pt. 13, a man with urothelial carcinoma. | *“Radiotherapy for bone metastases. What to do when the patient already received radiotherapy and the pain or loss of strength returns? The guideline is clear: impending spinal injury. In consultation with the radiotherapist, second radiotherapy is an option.”* | Future problems and treatment options in the physical domain. |
| Group 9 discussing pt. 24, a man with a brain tumour. | *“Home care presents scores from symptom diary, seems flat. Patient will undergo further diagnostics for experimental therapy. He is going to meet a buddy, but does not want to burden his friends with his illness. Still has problems structuring his days.”* | Current problems in the social domain. |
| Group 1 discussing pt. 118, a woman with dementia. | *“An aggressive woman with Alzheimer’s disease. Husband is depressed. […] Mental health care services are not accepted. Haloperidol is no longer given, as people want her to be lucid. Home care expects escalation.”* | Current and future problems in the psychological and social domain. |
| Group 7 discussing pt. 12, a man with urothelial carcinoma. | *“Comfortable and calm deathbed after a troubled sickbed. Transferred from hospital to hospice to home. Is this the way it should have gone?”* | Evaluation of care in the practical domain. |
| Group 2 discussing pt. 8, a woman with breast carcinoma. | *“Stable but poor situation. Mrs wants palliation or euthanasia and no further diagnostics in the hospital. The tumour marker has risen and progression is likely. Strongly desires autonomy. Trembling lower leg is a manageable problem at the moment. Clonazepam or diazepam are options.”* | Current problems, treatment options and wishes in the physical and existential domain. |
| Group 10 discussing pt. 67, a man with lung carcinoma. | *“Patient with lung carcinoma, in denial. Nurse specialist in mental care was involved, she is theologian and expert in existential problems. No continued conversation as of yet, patient is not up for it. There is a lot of anxiety in the patient. Discussed again, GP stays in touch and this is acceptable for the patient. Discussing the end of life is still not possible. Sometimes talking about death is not possible, and we just keep on caring as well as we can.”* | Current problems, treatment options and wishes in the existential domain. |
| Group 6 discussing pt. 4, a man with prostate and colon carcinoma. | *“Family was unsatisfied with the home care organization, chose another organization. Patient developed a delirium in the final days, did not want midazolam. In the final hours this was provided by the out-of-hours GP services.”* | Evaluation of care in the psychological domain. |
| Group 2 discussing pt. 28, a man with heart failure. | *“Terminal heart failure. What if a statement of terminal illness is given, and the patient does not die? 24 hours care is provided for a maximum of six months. One option is to anonymously inquire at health care insurer. Besides, a statement of terminal illness is required for hospice care.”* | Future problems in the practical domain. |
